# Position of the ISTENT Inject^®^ Trabecular Micro-Bypass System Visualized with the NIDEK GS-1 Gonioscope—A Postoperative Analysis

**DOI:** 10.3390/jcm12165171

**Published:** 2023-08-08

**Authors:** Julian Alexander Zimmermann, Jens Julian Storp, Ralph-Laurent Merté, Peter Heiduschka, Nicole Eter, Viktoria Constanze Brücher

**Affiliations:** Department of Ophthalmology, University of Muenster Medical Center, 48149 Muenster, Germany; jensjulian.storp@ukmuenster.de (J.J.S.); ralph-laurent.merte@ukmuenster.de (R.-L.M.); peter.heiduschka@ukmuenster.de (P.H.); viktoria.bruecher@ukmuenster.de (V.C.B.)

**Keywords:** glaucoma, MIGS, gonioscopy

## Abstract

Glaucoma is one of the leading causes of irreversible blindness globally and is characterized by the gradual loss of retinal ganglion cells. The primary risk factor for the development and progression of glaucoma is increased intraocular pressure (IOP). Numerous surgical interventions exist to lower IOP should conservative therapy fail. One trend in recent years has been minimally invasive glaucoma surgery (MIGS) as an alternative to traditional methods. The ISTENT inject^®^ is an ab interno trabecular micro-bypass implant designed to be implanted through the trabecular meshwork into the Schlemm’s canal to lower IOP. The aim of the study was the postoperative visualization and description of the positioning of the ISTENT inject^®^ using automated circumferential goniophotography. Patients with symptomatic cataracts and mild to moderate primary open-angle glaucoma (POAG), pseudoexfoliation glaucoma (PEX), and pigment-dispersion glaucoma were included who underwent combined cataract surgery with the ISTENT inject^®^ and received postoperative automated gonioscopy with the NIDEK Gonioscope GS-1 to visualize the location of the implant. Twenty-four implants of 14 eyes in 11 patients could be visualized. Out of the implants, 14.3% were in the trabecular meshwork, 46.4% were at the border between the trabecular meshwork and scleral spur, 25% were below the trabecular meshwork, and 14.3% of the implants were not detectable in the gonioscopy. In the overall cohort, a statistically significant IOP reduction was found over the 12-month postoperative observation period. Even in three eyes, in each of which both stents were located below the trabecular meshwork, an IOP reduction over 12 months was observed compared to the baseline IOP. In this study, vertical two-dimensional positioning of the ISTENT inject^®^ was performed for the first time using NIDKE GS-1 automated 360° goniophotography. The method is suitable for postoperative visualization, control, and documentation of positioning after ISTENT inject^®^ implantation. Further studies are needed to analyze the correlation between positioning of the ISTENT inject^®^ in the chamber angle and postoperative IOP reduction.

## 1. Introduction

Glaucoma is one of the leading causes of irreversible blindness worldwide and is characterized by the progressive loss of retinal ganglion cells. Elevated intraocular pressure (IOP) is the main risk factor for the development and progression of the disease. The most common method of treating glaucoma is to lower the IOP to prevent glaucomatous optic neuropathy. In first-line therapy, IOP-lowering eye drops and laser surgery are commonly used [1,2,3].

For several years, a current trend in glaucoma surgery has been a variety of minimally invasive glaucoma surgeries (MIGS) to lower IOP. The aim of MIGS is to provide an alternative to traditional methods, such as trabeculectomy, especially for patients with mild to moderate glaucoma damage. Surgical techniques and implants with a lower risk profile should lead to a comparable postoperative outcome. The goals are a shorter operation time, tissue-sparing procedures, comparatively simple handling, a lower risk profile, and a shorter rehabilitation time [4].

The ratio of the production and outflow of aqueous fluid in the anterior chamber is decisively responsible for IOP. Aqueous humor is produced by the epithelium of the ciliary body and flows mainly via the trabecular meshwork and Schlemm’s canal into the episcleral veins.

The ISTENT inject^®^ (Glaukos Corporation, Laguna Hills, CA, USA) is an ab interno trabecular micro-bypass implant designed to be implanted through the trabecular meshwork into the Schlemm’s canal to lower IOP. The ISTENT inject^®^ Trabecular Micro-Bypass System is approved for use in conjunction with cataract surgery for the reduction of IOP in adult patients with mild to moderate open-angle glaucoma [5]. The aqueous humor drains directly from the anterior chamber through the ISTENT inject^®^ into the Schlemm’s canal, bypassing the trabecular meshwork [6]. Two ISTENT injects^®^ are usually implanted after cataract surgery with the help of a preloaded injector into the trabecular meshwork. The implants are usually implanted two to three clock hours apart. Glaukos developed a 3rd generation implant, the ISTENT inject^®^ W, to achieve easier and better positioning of the stent during operation [6,7].

Scientific studies have shown the efficacy of the ISTENT inject^®^. Clinical trials have demonstrated significant reductions in IOP following the procedure, with a favorable safety profile. Patients who undergo ISTENT inject^®^ implantation often experience a decrease in their reliance on glaucoma medications [8,9,10].

Gonioscopy describes the examination of the iridocorneal angle (ICA), through which the main part of the aqueous humor is drained. The examination allows assessment of the ICA in terms of the peripheral anterior chamber depth to distinguish between open and closed angles, its pigmentation, abnormalities, and the configuration of the iris, especially in glaucoma patients, allowing for tailored treatment plans. Anatomical landmarks that aid in differentiation include the Schwalbe line, nonpigmented and pigmented trabecular meshwork, scleral spur, and ciliary body band.

In the age of MIGS, preoperative, intraoperative, and postoperative gonioscopy are of high importance. Preoperatively, gonioscopy helps select patients in whom implantation is possible due to anatomical conditions. Intraoperatively, gonioscopy is used to implant the devices in the chamber angle under visualization and postoperatively to assess the position of the implants.

The NIDEK Gonioscope GS-1 offers the possibility of automated ICA color photography using a 16-mirror-coated faceted gonioprism. This gonioscope is the first to automatically take 360° images of the chamber angle. For each facet of the gonioprism, 17 images were taken, each with a different focus. 

To our knowledge, this is the first study to graduate the positioning of the ISTENT inject^®^ based on anatomical landmarks in the chamber angle using 360° ICA photography.

## 2. Materials and Methods

The study was conducted at the Department of Ophthalmology at the University of Muenster Medical Center, Germany. Patients diagnosed with symptomatic cataracts and mild to moderate primary open-angle glaucoma (POAG), pseudoexfoliation (PEX) glaucoma, and pigment-dispersion glaucoma were included in the study. In this study, the positions of 28 stents implanted in 14 eyes in 11 patients were assessed in total. The patient population characteristics are summarized in Table 1. The exclusion criteria included reasons other than glaucoma that might cause abnormalities of the optic nerve head (e.g., optic disc drusen, neovascularization of the optic nerve head, tilted discs, and vascular occlusions). If ophthalmic examination was insufficient to establish such a connection, further testing, including neurologic examinations and MRI imaging, was performed. Further exclusion criteria were the presence of narrow-angle glaucoma, preoperative abnormalities of the chamber angle on gonioscopy, previous surgical IOP-lowering procedures, poor anterior chamber vision (e.g., corneal changes), and age under 18 years. The participants of the study underwent a standardized ophthalmic examination, including a refractive eye exam, the examination of the anterior segment, funduscopy, Goldmann applanation tonometry, perimetry using the automated Humphrey visual field analyzer (HFA II, model 750, Carl Zeiss Meditec AG, Jena, Germany) with the standard program of the 30–2 Swedish interactive threshold algorithm (SITA fast), fundus imaging of the optic disc (VISUCAM, Zeiss, Jena, Germany), and optical coherence tomography (OCT) of the optic nerve head and retinal nerve fiber layer (RNFL) using the spectralis OCT diagnostic imaging platform (Heidelberg Engineering GmbH, Heidelberg, Germany). A gonioscopy was performed preoperatively to confirm an open chamber angle, which appeared suitable for implantation of an ISTENT inject^®^. An automatic 360° gonioscopy was performed postoperatively using the NIDEK Gonioscope GS-1 (NIDEK, Gamagori, Aichi, Japan) to assess the position of the ISTENT inject^®^. Before the examination, the patient was given a drop of 0.5% proparacaine (Proparakain-POS, proxymetacaine hydrochloride, URSAPHARM, Saarbrücken, Germany) in the affected eye for local anesthesia. An ophthalmic gel (VIDISIC, Carbomer 980 2 mg/1 g, BAUSCH + LOMB, Berlin, Germany) was then applied to the gonioprism to create a space between the multimirror prism and the cornea. The prism did not touch the eye during the examination. A 360° image of the chamber angle was taken automatically by rotating the internal optical system while the patient fixated on a fixation target in the center of the prism. A total of 16 directions captured in 17 different foci were imaged. Depending on the anatomy, the image was taken after the examiner manually opened the eyelids.

**Figure 1:**
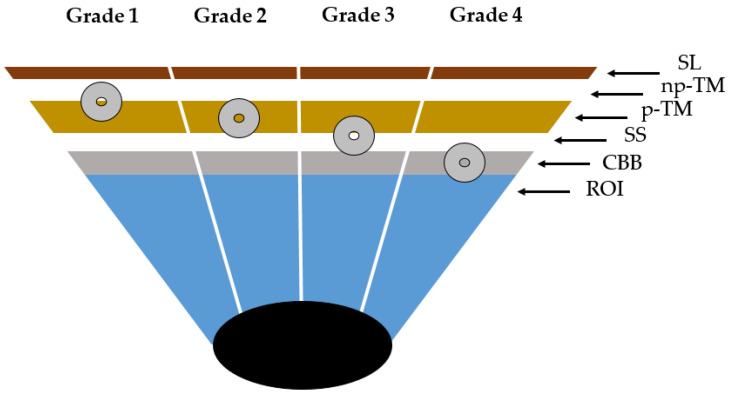
Position of the ISTENT inject^®^ in the chamber angle. Grade 1 refers to the central positioning of the implant in the nonpigmented and pigmented trabecular meshwork. Grade 2 is inferior: the lumen of the stent is at the level of the pigmented trabecular meshwork. In grade 3, the lumen of the stent lies in the transition zone between the pigmented trabecular meshwork and the scleral spur. Below the scleral spur, the stent lies at the level of the ciliary body band (grade 4). (SL, Schwalbe’s line; np-TM, nonpigmented trabecular meshwork; p-TM, pigmented trabecular meshwork; SS, scleral spur; CBB, ciliary body band; ROI, root of iris).

The images could be displayed as a single image or as a composite 360° image. The examiner selected the focus after the image had been taken. The images were selected by two experienced glaucoma specialists. The aim was to clearly show the anatomical landmarks (Schwalbe’s line, nonpigmented trabecular meshwork, pigmented trabecular meshwork, scleral spur, ciliary body band, root of iris of the chamber angle). The grading was done using images with a clear focus or images that were slightly blurred, but where the positioning of the stent could be made based on the distinguishable structures of the anterior chamber angle [11]. In the second step, the position of the ISTENT inject^®^ was graded based on these landmarks (Figure 1). If the examiners were unsure about the positioning of the ISTENT inject^®^ in the chamber angle, a third examiner was consulted. This retrospective, monocentric study was performed in accordance with the ethical standards issued by the ethics committee of the Medical Association of Westfalen-Lippe and the University of Münster; it also adhered to the tenets of the Declaration of Helsinki.

### 2.1. Device

The implantation of the ISTENT inject^®^ was performed in combination after cataract surgery, either under local or general anesthesia. The operations were performed by two experienced glaucoma surgeons. The ISTENT inject^®^ is a heparin-coated, non-ferromagnetic titanium stent with a diameter of 230 µm and a height of 360 µm. The diameter of the central lumen is 80 μm. At the head of the implant, there are four side flow outlets, each with a diameter of 50 µm. An injector loaded with two stents places the ISTENT inject^®^ in the chamber angle. Implantation through the trabecular meshwork into Schlemm’s canal increases aqueous outflow (average 2.5 µL/min).

The position for the device was 8 o’clock and 10 o’clock in the right eye and 2 o’clock and 4 o’clock in the left eye. During IOL implantation, visoelastic was placed in the anterior chamber to create sufficient space for implantation. A gonioprism allowed sufficient visualization of the chamber angle. The ISTENT inject^®^ injector was introduced into the anterior chamber via the incision and guided over the pupillary rim. The trocar tip was held perpendicular to the trabecular meshwork via the scleral spur. There was slight dimpling with the tip of the implant in the chamber angle. For implantation, the release button was pressed. The second stent was implanted 60–90 degrees apart. Postoperative treatment consisted of a topical combination of 5.0 mg/mL gentamicin sulfate and 1.0 mg/mL dexamethasone sodium phosphate eyedrops (dexagentamicin eye drops; URSAPHARM, Saarbrücken, Germany) four times a day and 0.3 mg/g dexamethasone and 5.0 mg/g gentamicin sulfate eye cream (dexagentamicin eye cream; URSAPHARM, Saarbrücken, Germany) at night for one month. All glaucoma medications were stopped at the time of surgery.

Postoperative controls at the Department of Ophthalmology at the University Hospital Münster were performed after one month, six months, and one year (Table 2).

### 2.2. Statistical Analysis

Data were collected from the electronic patient records (FIDUS, Arztservice Wente GmbH, Darmstadt, Germany) and statistical analysis was performed using GraphPad Prism 10 (GraphPad Software, Boston, MA, USA). Normal distribution was tested using the Shapiro–Wilk test. We used the Kruskal–Wallis test followed by the Dunn’s multiple comparisons test. A *p*-value of less than 0.05 was considered statistically significant.

## 3. Results

**Figure 2:**
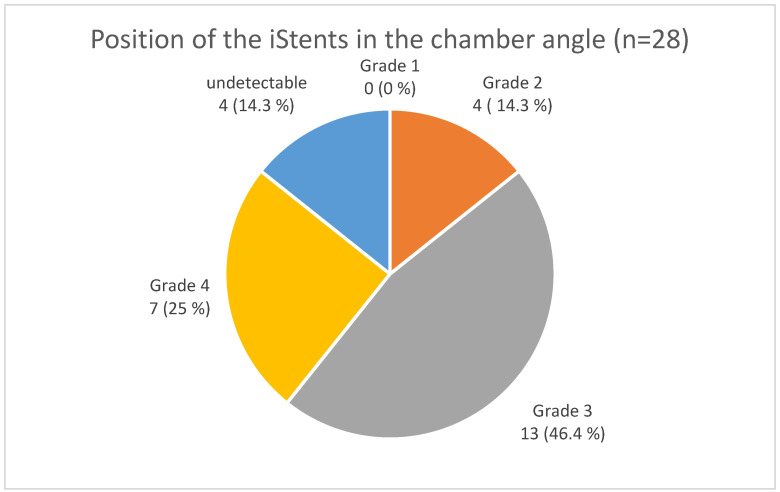
Position of the ISTENT inject^®^ in the chamber angle (*n* = 28).

Out of the 28 implanted stents, four (14.3%) were undetectable using automated gonioscopy. A grade 2 position was found in four cases, grade 3 in 13 cases, and grade 4 in seven cases (Figure 1 and Figure 2).

In three eyes, stents were found to be all grade 2 and 3 (group 1). In three other eyes, only grade 4 stents were found (group 2). Both groups showed reductions in pressure over the 12-month observation period. In group 1, the baseline IOP was 16.83 mmHg (±2.75 mmHg), the IOP at 1 month was 14 mmHg (±1.41 mmHg), at 6 months it was 13 mmHg (±5.66 mmHg), and at 12 months it was 14 mmHg (±2.82 mmHg). In group 2, the baseline IOP was 18.67 mmHg (±3.06 mmHg), the IOP at 1 month was 11.67 mmHg (±2.52 mmHg), at 6 months it was 15.50 mmHg (±6.36 mmHg), and at 12 months it was 16 mmHg (±2.82 mmHg).

## 4. Discussion

To the best of our knowledge, this is the first in vivo study to analyze the vertical positioning of the ISTENT inject^®^ in the chamber angle using automated gonioscopy in high-resolution images captured by the NIDEK GS-1 Gonioscope.

**Figure 3:**
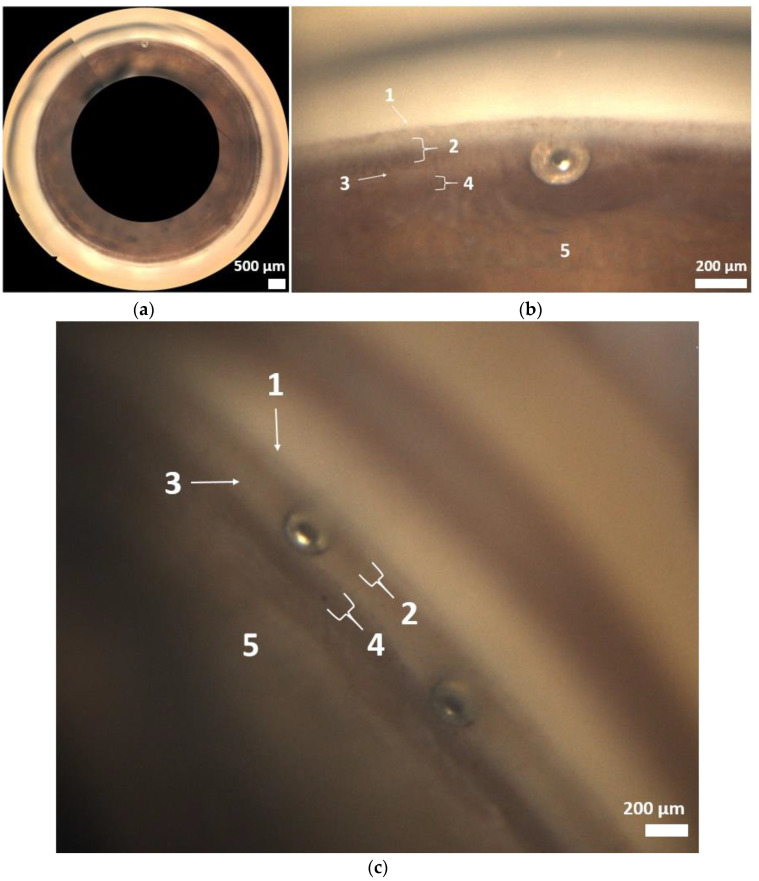
(**a**): 360° gonioscopy with the ISTENT inject^®^. (**b**,**c**): Position of ISTENT inject^®^ in the chamber angle (1 Schwalbe’s line; 2 nonpigmented and pigmented trabecular meshwork; 3 scleral spur; 4 ciliary body band; 5 root of iris).

The analysis was based on the anatomical landmarks of the chamber angle (Figure 1 and Figure 3a–c). In most cases (Grade 2 and 3 in 60.7%), implantation was performed as intended in the trabecular meshwork region (Figure 2). Across all implants, there was a statistically significant reduction in IOP at one month, six months, and one year postoperatively (Table 2).

MIGS devices are specifically designed to reduce IOP in patients with mild or moderate glaucoma and to minimize the risk of complications associated with more invasive glaucoma procedures. MIGS has revolutionized the field of glaucoma management [4]. The Glaukos ISTENT inject^®^ is considered a safe and effective method for IOP reduction with few complications and a low rate of adverse events in the postoperative course [6].

The ISTENT inject^®^ is the smallest device that can be implanted in the human body, made of heparin-coated, non-ferromagnetic titanium. Bypassing the trabecular meshwork facilitates aqueous humor drainage and lowers IOP. A combination treatment strategy is offered by the ISTENT inject^®^, which is usually implanted after cataract surgery [6].

In 2012, the first generation of the ISTENT^®^ was approved by the U.S. Food and Drug Administration (FDA) as the first MIGS device to be combined with cataract surgery. In 2018, the second generation of the ISTENT^®^, the ISTENT inject^®^, was approved, and in 2020, the ISTENT inject W^®^ was released. The iStent inject W^®^ is the third generation of the ISTENT^®^, characterized in particular by a wider flange [12].

Cataract surgery alone is considered to result in long-term IOP reduction. The mechanism behind IOP reduction through cataract surgery is still controversial [13,14,15]. Compression of Schlemm’s canal is assumed to follow an increase in lens volume with age, providing traction over the zonules and subsequently on the ciliary body. By replacing the natural lens in this way, the outflow of aqueous humor increases.

It is also speculated that modern-day phacoemulsification-induced inflammation through the release of cytokines and prostaglandins leads to a decrease in aqueous humor production and the improvement of aqueous outflow, analogous to the mechanism of action behind selective laser trabeculoplasty.

Furthermore, a short-term increase in pressure during cataract surgery may lead to increased patency in the trabecular meshwork and Schlemm’s canal. Despite the existence of all these theories, disagreement still exists on the potency, clinical importance, and long-term variability of each process [13,14,15].

Gonioscopy is a technique used to visualize and examine the iridocorneal angle (ICA) of the eye. Gonioscopy is part of a complete eye examination and therefore has both diagnostic and therapeutic implications [16]. Understanding the anatomy of the chamber angle is essential for the diagnosis and management of various ocular conditions, particularly in glaucoma patients. In glaucoma patients, gonioscopy helps differentiate between open-angle glaucoma (OAG) and angle-closure glaucoma (ACG). Furthermore, gonioscopy provides information about the grade of the pigmentation of the chamber angle and abnormalities, such as synechiae, neovascularization, and angle recession [17]. In OAG, different pathomechanisms, visible in gonioscopy, cause a reduced outflow of aqueous humor via the trabecular meshwork.

In pigment-dispersion glaucoma, contact between the posterior iris and the anterior ciliary zonules results in the release of pigment, particularly into the anterior chamber [18]. The pigment settles in the trabecular meshwork and can thus reduce aqueous humor outflow, leading to an increase in IOP. Apart from iris transillumination and pigment on the corneal endothelium (Krukenberg spindle), pigment deposition is classically found in the trabecular meshwork. Furthermore, in both pigment-dispersion syndrome and pseudo-exfoliation syndrome, pigment can be found in the area of Schwalbe’s line or anterior to it in the form of the so-called Sampaolesi line. In pseudo-exfoliation syndrome, these are fibrillar deposits in the anterior segment of the eye [19].

Manual gonioscopy is highly dependent on the experience of the examiner to obtain accurate and reliable results. Manual gonioscopy proficiency requires much practice and instruction. To improve the examiner’s abilities and guarantee reliable assessments, it is essential to evaluate a wide range of patients from various angles [20].

Only part of the ICA can be examined at any one time. The duration of the examination and the visualization of the chamber angle highly depend on the patient’s cooperation. The interpretation of the findings collected during manual gonioscopy is subjective, as different examiners may perceive and grade angle structures differently. This can lead to inconsistent diagnoses and treatment decisions, particularly when multiple healthcare professionals are involved in the patient’s care.

There is no routine photo documentation in the form of images, so interpretation is the sole responsibility of the examiner. Both investigation and documentation use time resources [21]. Newly introduced automatic gonioscopy allows the acquisition of a 360° image of the chamber angle and records it for later analysis. The focus of the images varies automatically so that all structures in the chamber angle, such as implanted MIGS, can be captured [21,22,23].

With a variety of new MIGS devices that drain aqueous humor from the anterior chamber into the canal of Schlemm through implantation into the chamber angle, gonioscopy is once again important. Preoperatively, the technique is used to assess the chamber angle to determine whether implantation is possible regarding the Shaffer grading system, a system describing the extent to which the chamber angle is open. Postoperatively, gonioscopy allows for the analysis of the position of the implanted stents [4].

In previous work, GS-1 gonioscopy has been used to visualize the location of the Hydrus^®^ Microstent. The Hydrus^®^ Microstent belongs to the group of MIGS devices designed to bypass the trabecular meshwork. By implanting it in Schlemm’s canal, the Hydrus^®^ Microstent facilitates the outflow of aqueous humor and thus lowers IOP [24]. Laroche et al. were able to visualize five mispositioned Hydrus^®^ Microstents using automatic gonioscopy and, for the first time, make statements about their positioning in the chamber angle. Despite a less-than-ideal position of the Hydrus^®^ Microstent in the presented cases, IOP was lowered postoperatively after implantation [25].

Implantation of the ISTENT inject^®^ is considered a low-risk operation. A common complication is malpositioning of the stent. To date, there has been no uniform description of malpositioned stents.

Implantation should be performed through the trabecular meshwork to achieve aqueous humor outflow into Schlemm’s canal. The two implants should be placed at least two clock hours away from each other. Proper placement is defined as a visible flange in the anterior chamber [10]. The outflow via the trabecular meshwork and Schlemm’s canal behind it represents the conventional pathways of aqueous humor. Approximately 10% of aqueous humor drains via the uveoscleral pathway through the intracellular space of the ciliary muscle cells and iris vessels [26].

Different techniques of characterization of the anterior chamber, such as gonioscopy and anterior segment optical coherence tomography (AS-OCT), can help in postoperative evaluation.

Gillmann et al. investigated the positioning of the ISTENT inject^®^ miccrostent in the chamber angle in 2019 and in a follow-up study in 2020. They highlight AC-OCT to locate and describe ISTENTs^®^, especially those that cannot be found by gonioscopy. High-resolution pictures of the anterior segment features, including the cornea, iris, and anterior chamber angle, are obtained using AS-OCT.

Furthermore, AS-OCT can detect implants deep in the chamber angle that appear well-positioned on gonioscopy. Deep implantation of the microstent within or behind the trabecular meshwork can result in the implants being partially invisible on gonioscopy. At this point, AS-OCT can be used for visualization, especially in first- and second-generation ISTENTs with a smaller flange [27,28]. Fernándes-Barrientos et al. describe malpositioned stents in 18% of 34 cases after implantation of the ISTENT inject^®^. In all of these cases, a satisfactory reduction of IOP was found, so no additional intervention was necessary [29].

In our study cohort, 14.3% of the implanted ISTENT injects^®^ were found in the area of the pigmented trabecular meshwork (Grade 2). Most of the implants (46.6%) also had contact with the scleral spur (Grade 3). The scleral spur is a shelf-like structure of the sclera composed of elastic and collagenous fibers and myofibroblasts. Anteriorly, the scleral spur is bounded posteriorly by the trabecular meshwork and by the ciliary muscle. Presumably, its function is to facilitate aqueous humor outflow by keeping Schlemm’s canal open [30].

Although 25% of the implants were positioned below the trabecular meshwork (Grade 4), a significant reduction in IOP was found across all patients over the one-year observation period. Even in three eyes, in each of which both stents were located below the trabecular meshwork, an IOP reduction over 12 months was observed compared to the baseline IOP. This is interesting considering that the reduction in IOP is thought to occur primarily via improved aqueous humor outflow through the implant via the trabecular meshwork and Schlemm’s canal. In our opinion, there are several possible explanations for this: 

An assessment of the positioning into the depth of the chamber angle is not possible with two-dimensional imaging alone. The lumen of the implant may be in contact with the Schlemm’s canal despite vertical positioning outside the trabecular meshwork. Furthermore, the lumen of the implant is centrally located in the flange, so contact of the flange with structures located outside the trabecular meshwork does not necessarily indicate mispositioning. 

An accurate assessment of implant positioning based on anatomical landmarks is not always clear, depending on anatomical conditions, such as the degree of pigmentation of the chamber angle and the degree to which the angle is open.

In addition, two ISTENT injects^®^ are implanted, so that an incorrectly positioned stent can possibly be compensated for by the second implant. At this point, subgroup analyses for the analysis of two differently positioned stents are planned, with higher case numbers in the future.

Therefore, the limitations of the study are the small number of patients and implanted ISTENTs^®^ that could be investigated using the GS-1. Further studies with a larger number of cases are necessary to demonstrate any effects of the position of the ISTENT inject^®^ in the chamber angle on the postoperative outcome. In this context, subgroups should be formed according to the position of the stents in the chamber angle to draw conclusions on the relationship between position and IOP development. Furthermore, according to Gillmann et al., the performance of an AC-OCT examination complements the diagnostics for characterizing the position of the ISTENT inject^®^, especially if these cannot be depicted using gonioscopy [27]. Furthermore, we also consider AC-OCT useful for postoperative evaluation of the location of those ISTENT injects^®^ that may be in contact with Schlemm’s canal by tilting in depth, although they appear mispositioned in a two-dimensional view.

## 5. Conclusions

Our study establishes 360° automated gonioscopy as a reliable and practical technique to visualize ISTENT injects^®^ within the iridocorneal angle. For postoperative characterization of the position after ISTENT inject^®^ implantation, we see automatic gonioscopy as a suitable and descriptive method in addition to classical gonioscopy and AS-OCT. Further studies are recommended to establish a possible connection between the degree of positioning of the ISTENT inject^®^ in the chamber angle and the postoperative course.

## Figures and Tables

**Table 1:** Demographics and baseline characteristics of the study cohort. Data on continuous variables are reported as the mean (±standard deviation) or the median (25th percentile; 75th percentile), depending on the data distribution.

Patients/Eyes/ISTENT Inject^®^ (*n*)	11/14/28
Diagnosis	
POA-Glaucoma	7/11 (63.6%)
PEX-Glaucoma	4/11 (36.4%)
Age (years)	70.00 [64.75; 74.00]
Gender (m/f)	4 (36.4%)/7 (63.6%)
Visual field MD (dB)	−7.25 [−12.84; −3.90]
Baseline IOP (mmHg)	17.57 ± 3.57
Number of antiglaucoma eye drops at baseline	3.45 ± 1.63

(*n*, number; POA glaucoma, primary open-angle glaucoma; PEX glaucoma, pseudo-exfoliative glaucoma; m, male; f, female; IOP, intraocular pressure; MD, mean deviation; dB, decibel).

**Table 2:** **IOP and anti-glaucomatous eye drops during the observation period.** Data on continuous variables are reported as the mean (±standard deviation) or the median (25th percentile; 75th percentile), depending on the data distribution.

IOP in mmHg	Baseline versus postoperative assessment	17.57 ± 3.57	*p*-value
	1 month	12.83 ± 1.75	0.0043
	6 months	12.88 ± 3.82	0.0073
	12 months	13.33 ± 1.80	0.0286
Daily antiglaucomatous eye drops (*n*)	Baseline versus postoperative assessment	3.45 ± 1.63	*p*-value
	1 month	1.75 ± 1.60	0.0936
	6 months	2.88 ± 1.00	>0.9999
	12 months	3.00 ± 1.00	>0.9999

(IOP, intraocular pressure; *n*, number).

## Data Availability

Not applicable.
